# CCR6 is essential for effective immunity against *Mycobacterium tuberculosis* infection in mice

**DOI:** 10.1128/iai.00154-26

**Published:** 2026-04-30

**Authors:** Summer J. Harris, Oyindamola O. Adefisayo, Rachel K. Meade, E. Ashley Moseman, Charlie J. Pyle, Clare M. Smith

**Affiliations:** 1 Department of Molecular Genetics and Microbiology, Duke University 12277 , Durham, North Carolina, USA; 2 University Program in Genetics and Genomics, Duke University 349027, Durham, North Carolina, USA; 3 Department of Integrative Immunobiology, Duke University 3065, Durham, North Carolina, USA; University of California Davis, Davis, California, USA

**Keywords:** tuberculosis, immunity, mouse models, granuloma, cytokines, CCR6

## Abstract

Tuberculosis (TB) is a global epidemic that has threatened human health throughout recorded history. TB disease, caused by infection with *Mycobacterium tuberculosis* (*M.tb*)*,* is heterogeneous between individuals, and clinical TB outcomes are impacted by genetic and environmental risk factors. *M.tb*-infected individuals must maintain a careful balance of cytokines and chemokines to eliminate or contain the bacteria without causing excessive inflammation and lung damage. The CC chemokine receptor 6 (CCR6) is expressed by several immune cell populations that are classically involved in the host response to *M.tb*. However, the precise functions of CCR6 in the context of TB disease pathogenesis remain underexplored. Here, we show that mice lacking the CCR6 receptor (CCR6 KO) failed to restrict bacterial burden in the lungs, leading to increased dissemination compared to wild-type C57BL/6J (B6) mice, with a more pronounced effect in the lungs of females. CCR6 KO mice also developed necrotic pulmonary lesions that were phenotypically distinct from B6 mice and produced elevated levels of pro-inflammatory cytokines and chemokines at the onset of adaptive immunity, particularly IL-17. Long-term infection experiments revealed that the absence of CCR6 enhances risk for mortality following *M.tb* infection, particularly in females. This study provides insights into the role of CCR6 during TB pathogenesis and establishes its importance in maintaining protective immunity against *M.tb* within the context of a genetically tractable mouse model that forms necrotic pulmonary granulomas.

## INTRODUCTION

An estimated 10.8 million people worldwide were infected with tuberculosis (TB) in 2023, and among those cases, 1.25 million people succumbed to disease (1); 90% of people who become infected with *Mycobacterium tuberculosis* (*M.tb*), the causal agent of TB, successfully control infection, while the other 10% progress to an active disease state, usually as a result of a failure of the host’s immune responses (2). Effective host resistance to *M. tb* necessitates coordinated innate and adaptive immune responses (3). As such, numerous leukocyte effector cells involved in both the innate and adaptive response, such as neutrophils and T cells, are recruited to the site of infection where they play diverse roles necessary for bacterial containment and elimination (3, 4). Recruitment of these cell types is dependent on cytokines and chemokines (5–10). This is supported by a transcriptional profiling study of the innate immune response in mouse lung tissue following low-dose *M.tb* infection, which revealed that many cytokines and chemokines are strongly upregulated 12–21 days following infection (11) and are likely correlated with the recruitment of immune cell populations to the site of infection. Cytokine- and chemokine-mediated recruitment and activation of microbicidal immune cells are crucial for controlling bacterial growth but require balance with tolerogenic signaling to avoid causing excessive tissue injury (12), which may exacerbate disease outcomes in the host. In addition to direct microbicidal activity, recruited cells also participate in the formation of mycobacterial granulomas, which are organized aggregates of immune cells around infecting bacteria (13). Chemokines play a critical role in granuloma formation (12, 14) and may determine if the structure functions as a protective niche for the bacteria or as a host-defense mechanism (15).

Despite their importance, identifying the specific roles and impacts of individual cytokines and chemokines during infection remains challenging. This complexity arises from the fact that most cytokines and chemokines have multiple cellular targets and engage in intricate, redundant signaling pathways (12, 14). This makes single receptor-single cytokine/chemokine pairs particularly attractive for study as they provide a straightforward framework for detangling intricate patterns of cytokine and chemokine responses during TB infection. The CCR6 receptor and its only known ligand CCL20 are one such pair (16). CCR6 is expressed on immature dendritic cells (DC), innate lymphoid cells (ILCs), B cells, and T cells (17). The CCR6–CCL20 axis has been predominantly studied for its role in autoinflammatory conditions like inflammatory bowel disease (IBD) (17) and cancer (18) but has also been implicated in various infectious diseases such as human immunodeficiency virus (HIV) (19, 20), pneumococcal meningitis (21), and *Helicobacter pylori* gastritis (22, 23). CCL20 is upregulated in *M.tb*-infected monocytes (24), activated peripheral blood mononuclear cells (PBMCs) from human patients with active TB (25), and in mice infected via intratracheal route with the *Mycobacterium bovis*-derived vaccine strain Bacille Calmette-Guérin (BCG) (26). In both non-human primates (NHPs) (27) and human patients (28) with active TB, the majority of airway CD4^+^ T cells co-expressed CCR6 and another chemokine receptor CXCR3 (27, 28). IFN-γ production is a critical host defense response against *M.tb* (29–31). However, Proulx and colleagues (32) identified genetically diverse mouse strains that produce low levels of IFN-γ following infection and are also resistant to *M.tb* infection. Importantly, they found that bacterial control in these mice correlated strongly with elevated levels of CCR6 on CD4^+^ T cells. This is particularly relevant as CD4^+^ T cells can mediate mycobacterial control in both IFN-γ-dependent and IFN-γ-independent manners (33). Collectively, these data suggest that CCR6 is a prominent feature of the host adaptive immune response to *M.tb* infection that can operate independently of canonical IFN-γ-driven TB immunity and warrants further investigation.

To better understand the role of CCR6 in TB pathogenesis, we infected CCR6 receptor knockout (CCR6 KO) mice with aerosolized *M.tb* and compared disease phenotypes with co-infected B6 controls. In contrast to a previous study reporting that CCR6 is not required for the establishment of T cell-mediated immunity or bacterial clearance following infection with the attenuated BCG strain (26), we show that the CCR6 deficiency disrupts adaptive immune control of virulent *M.tb*, resulting in a survival defect. Unlike in B6 mice that do not form the organized *M.tb*-containing granulomas characteristic of human pulmonary TB (34), we identified multiple instances of organized necrotic lesions in infected CCR6 KO mice. These findings suggest that CCR6 signaling may play a role in shaping the pulmonary immune landscape in response to *M.tb*. Moreover, CCR6 KO mice may represent a novel and tractable system for investigating granuloma formation and dynamics following *M.tb* infection on a B6 background. This new mammalian *M.tb*-granuloma model has the potential to enhance our capacity to study granuloma biology *in vivo* and explore the impact of granulomas on TB outcome and the pharmacodynamics of investigational therapies.

## RESULTS

### CCR6 KO mice have elevated bacterial burden

**Fig 1:**
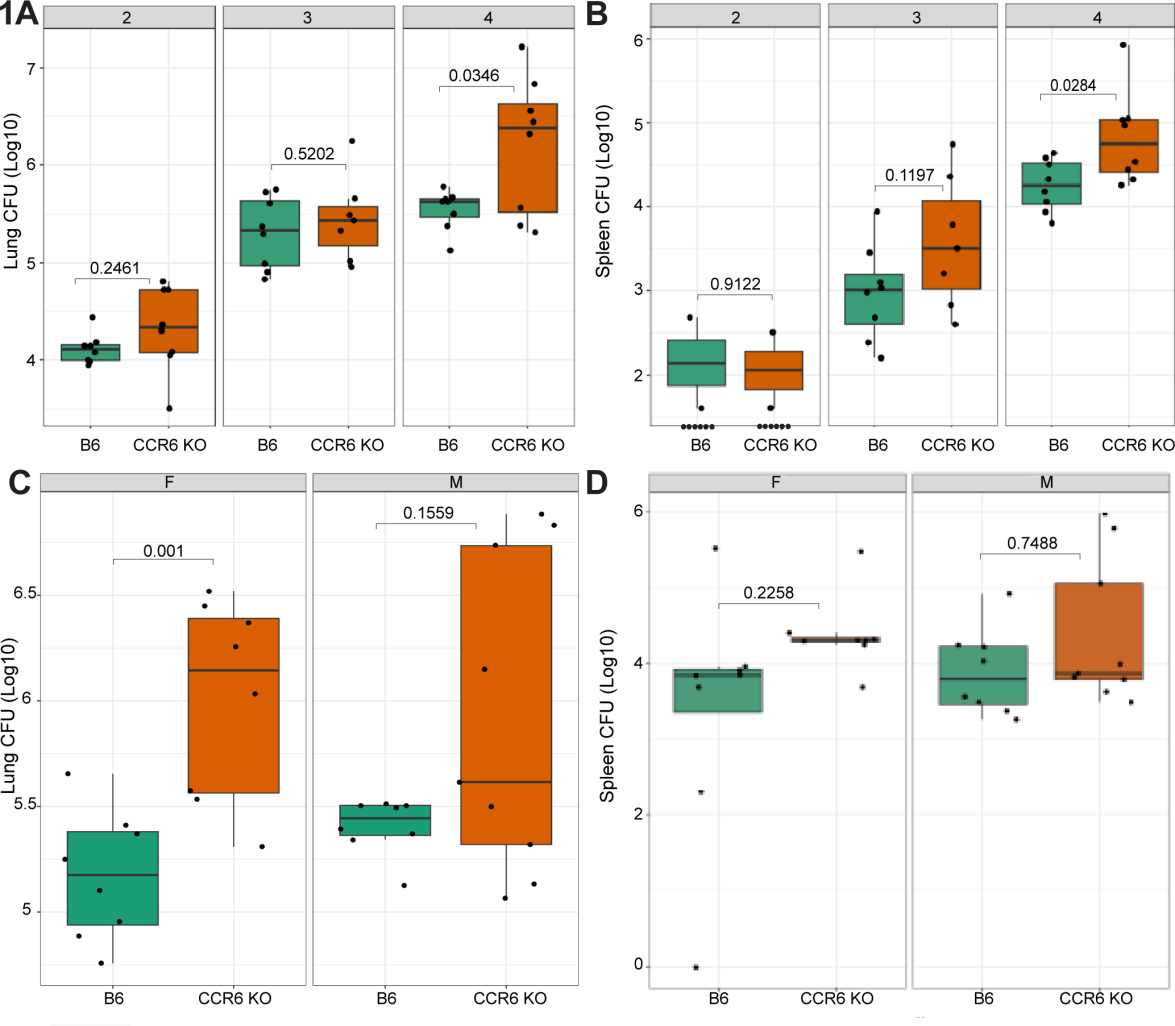
CCR6 KO mice have higher bacterial burden in the lung and spleen at 4 weeks post-infection. Bacterial burden measured from (**A**) lung and (**B**) spleen homogenate at 2, 3, and 4 weeks post-infection from CCR6 KO and B6 mice infected with *M.tb* H37Rv (*N* = 8 mice per strain; both sexes included in equivalent quantity). Bacterial burden levels, stratified by sex, at 4 weeks post-infection from (**C**) lung and (**D**) spleen (*n* = 8–9 mice per strain and sex, two independent cohorts). Hypothesis testing was performed by two-way analysis of variance (ANOVA) and Tukey’s *post hoc* test on batch-normalized log_10_-transformed values.

To investigate the role of CCR6 *in vivo* during *M.tb* infection, we infected CCR6 KO mice of both sexes via the aerosol route and compared their infection dynamics to B6 mice. We observed no significant differences at 2 or 3 weeks post-infection, prior to the onset of the adaptive immune response (Fig. 1A). However, CCR6 KO mice yielded significantly higher bacterial burdens from their lungs (Fig. 1A; *P* = 0.0346) and spleens (Fig. 1B; *P*=0.0284) around 4 weeks post-infection (35). Upon further analysis, we found that the burden differences in the lungs of CCR6 KO mice were sex-specific. The lungs of CCR6 KO females had significantly higher bacterial burdens relative to female B6 control lungs at 4 weeks post-infection (Fig. 1C; *P* = 0.0462). No significant lung burden difference was observed between CCR6 KO and B6 males (Fig. 1C; *P* = 0.373). Furthermore, we did not observe sex-specific differences in bacterial control in the spleen at 4 weeks post-infection (Fig. 1D).

### CCR6 KO mice develop necrotic lung lesions

**Fig 2:**
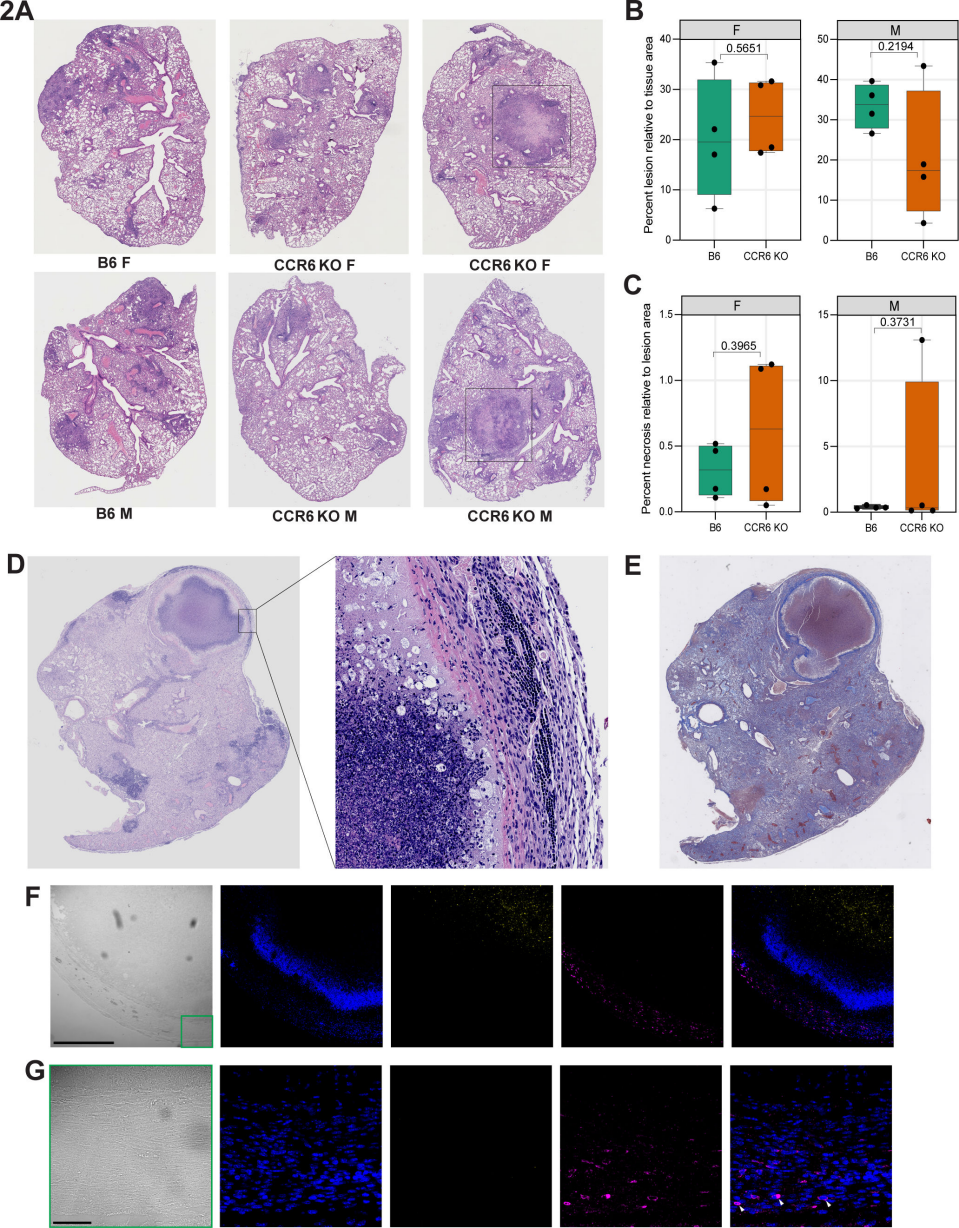
CCR6 KO mice develop necrotic lesions. (**A**) Representative whole-slide scans of H&E-stained lung sections from male and female CCR6 KO and B6 mice, collected at 4 weeks post-infection. Areas of organized lesion formation are indicated by a black box. Quantification of H&E-stained images demonstrating (**B**) the percent lesion area relative to total tissue area, and (**C**) the percent necrotic area relative to the quantified lesion area, with each dot representing an individual mouse at 4 weeks post-infection. For panels B and C, no significant difference could be detected by unpaired *t*-test with Welch’s correction. (**D**) Representative H&E-stained whole-lung section from a male CCR6 KO mouse at 15 weeks post-infection (left panel). The right panel is a 40× magnification of the boxed area from the left panel showing the periphery of a well-organized, necrotic lesion. (**E**) Masson’s trichrome-stained slide from the same representative sample in panel D, indicating the fibrotic capsule (deep blue stain) surrounding the lesion. Brightfield and immunofluorescence-stained images of a slide from the same sample showing cell nuclei DAPI (blue), neutrophil Ly-6G (yellow), and T cell CD4 (magenta) localization in necrotic granulomas at (**F**) 10× (scale bar 500 µm) and an inset (green box) (**G**) 63× magnification (scale bar 50 µm).

We next assessed the impact of CCR6 deficiency on lung pathology during *M.tb* infection. At 4 weeks post-infection, we observed two major pulmonary lesion phenotypes in the lungs of CCR6 KO mice. The first type was similar to lesions observed in *M.tb*-infected B6 mice (Fig. 2A, middle panel), which are characterized by disorganized clusters of macrophages and lymphocytes (36). The second type of lesion had greater structural complexity with stratified cellular lamina surrounding a necrotic core (Fig. 2A, right panel). We then assessed lung damage using an artificial intelligence (AI) model (as described in Materials and Methods) to quantify cellular infiltration and necrosis within the lungs. Using that model, we found no significant differences in lesion size relative to the entire lung area between CCR6 KO mice and B6 controls (Fig. 2B). We also did not observe a significant difference in areas of necrosis within the lesion between mouse lungs from CCR6 KO mice and B6 controls (Fig. 2C). However, a subset of CCR6 KO mice developed pulmonary lesions with prominently expanded areas of necrosis (Fig. 2A and C). Histopathological analysis of lungs from independent cohorts of male CCR6 KO mice at 10–15 weeks post-infection revealed that 30% (*n* = 3 of 10) of those animals formed well-organized, necrotic granulomas (Fig. 2D, left panel). These granulomas resembled mycobacterial granulomas found in human patients (37), NHPs (38), and zebrafish (39), with a well-organized periphery comprised of immune cells, including epithelioid macrophages and foamy macrophages, circumscribing a central zone of necrotic caseum (Fig. 2D, right panel). Masson’s trichrome staining revealed that *M.tb* granulomas in CCR6 KO mice are surrounded by a collagen-rich fibrotic layer (Fig. 2E), which is also present in mature human TB granulomas (40). Immunofluorescence staining of this second type of granuloma revealed little to no neutrophils in the cellular cuff but showed an abundance of the neutrophil marker protein Ly6G in the interior, acellular necrotic core (Fig. 2F and G, panel iii). However, CD4^+^ T cells were abundant in the outlying cellular cuff (Fig. 2F and G, panel iv). Interestingly, this granuloma type also showed outlying CD4^+^ T cell-rich lymphoid aggregates (Fig. S1) that are consistent with granuloma-associated lymphoid tissue found at the periphery of NHP and human *M.tb* granulomas (41–43).

### *M.tb*-infected CCR6 KO mice have elevated IL-17 levels

**Fig 3:**
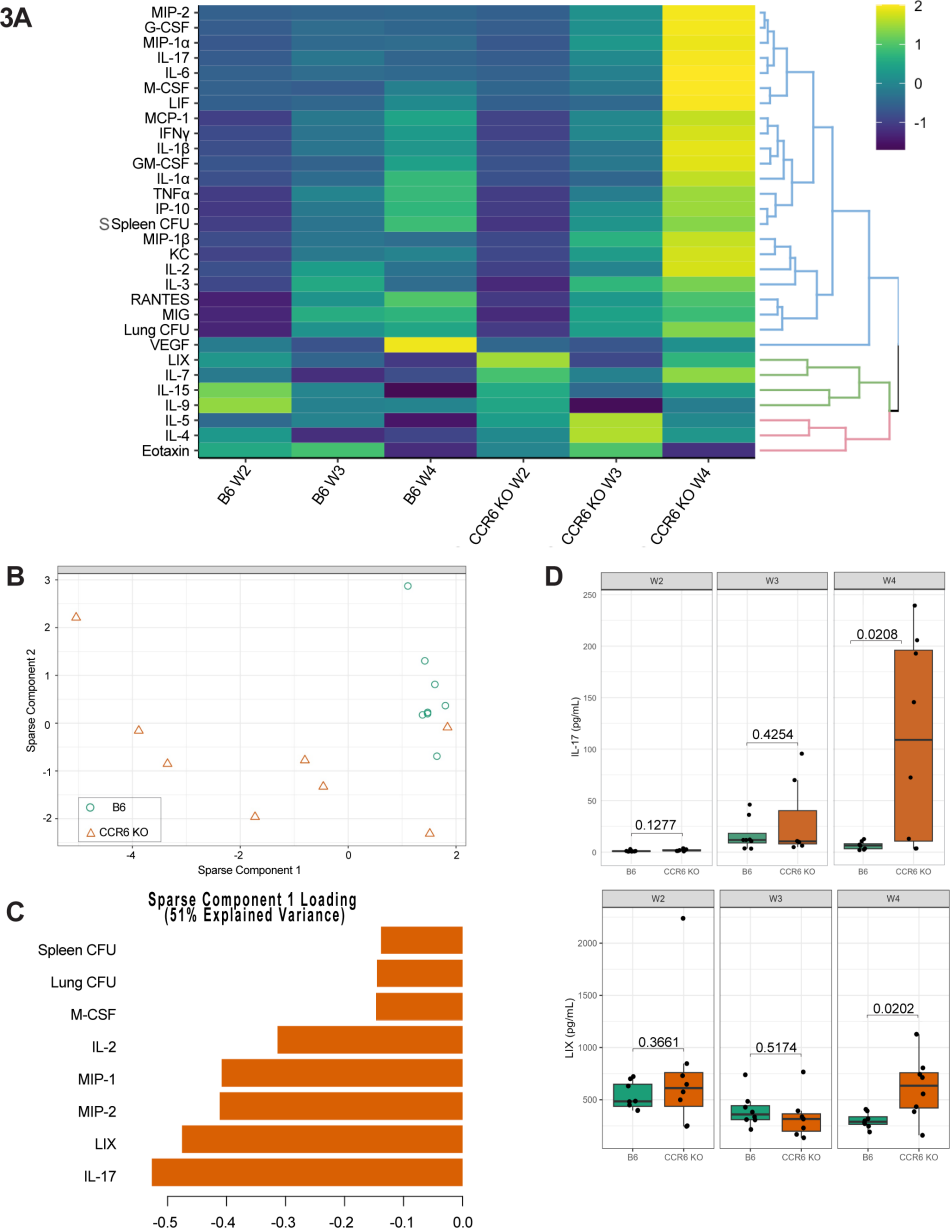
IL-17 and LIX levels dominate the immune responses in the lungs of *M.tb*-infected CCR6 KO and B6 mice. (**A**) Heatmap depicting scaled phenotypes at 2 weeks (W2), 3 weeks (W3), and 4 weeks (W4) post-infection, hierarchically clustered and divided into 3 k clusters. (**B**) Individual mice plotted against the first two sPLS-DA components, which explained the greatest variance in the data from measurements collected 4 weeks post-infection. (**C**) Phenotype loadings contributing to the first sparse component with a 51% explained variance. (**D**) IL-17 and LIX levels measured from lung homogenate by multiplex ELISA at 2, 3, and 4 weeks post-infection. Hypothesis testing was performed by two-way analysis of variance (ANOVA) and Tukey’s *post hoc* test.

After identifying CCR6 as a determinant of infection-associated lung pathology, we evaluated the role of CCR6 deficiency on pulmonary inflammation in *M.tb*-infected mice. We conducted immunological profiling on lung homogenate from *M.tb*-infected CCR6 KO mice and B6 controls via multiplex ELISA. At 4 weeks post-infection, CCR6 KO mice produced elevated levels of numerous cytokines and chemokines, including inflammatory cytokines IL-2 and IL-17, neutrophil/macrophage maturation cytokines M-CSF, G-CSF, and GM-CSF, as well as the neutrophil attractant chemokines KC (CXCL1), LIX (CXCL5), MIP-1α, MIP-1β, and MIP-2 (CXCL2) (Fig. 3A). Conversely, CCR6 KO mice produced lower levels of the angiogenesis signaling protein vascular endothelial growth factor (VEGF), which has been shown to have CCR6-dependent expression in solid tumors (44, 45). To identify unique features of CCR6-dependent inflammation, we conducted sparse partial least squares discriminant analysis (sPLS-DA) across lung cytokine and chemokine levels as well as lung and spleen burden measurements from CCR6 KO and B6 mice at 4 weeks post-infection (Fig. 3B). IL-17 and CXCL5 were identified as the strongest features underlying sparse component 1, which explained 51% of the variance between groups (Fig. 3C). Host genotype-associated differences in IL-17 and CXCL5 were only observed at the 4-week time point (Fig. 3D) and were not impacted by sex.

### *M.tb* susceptibility in CCR6 KO mice is sex-dependent

**Fig 4:**
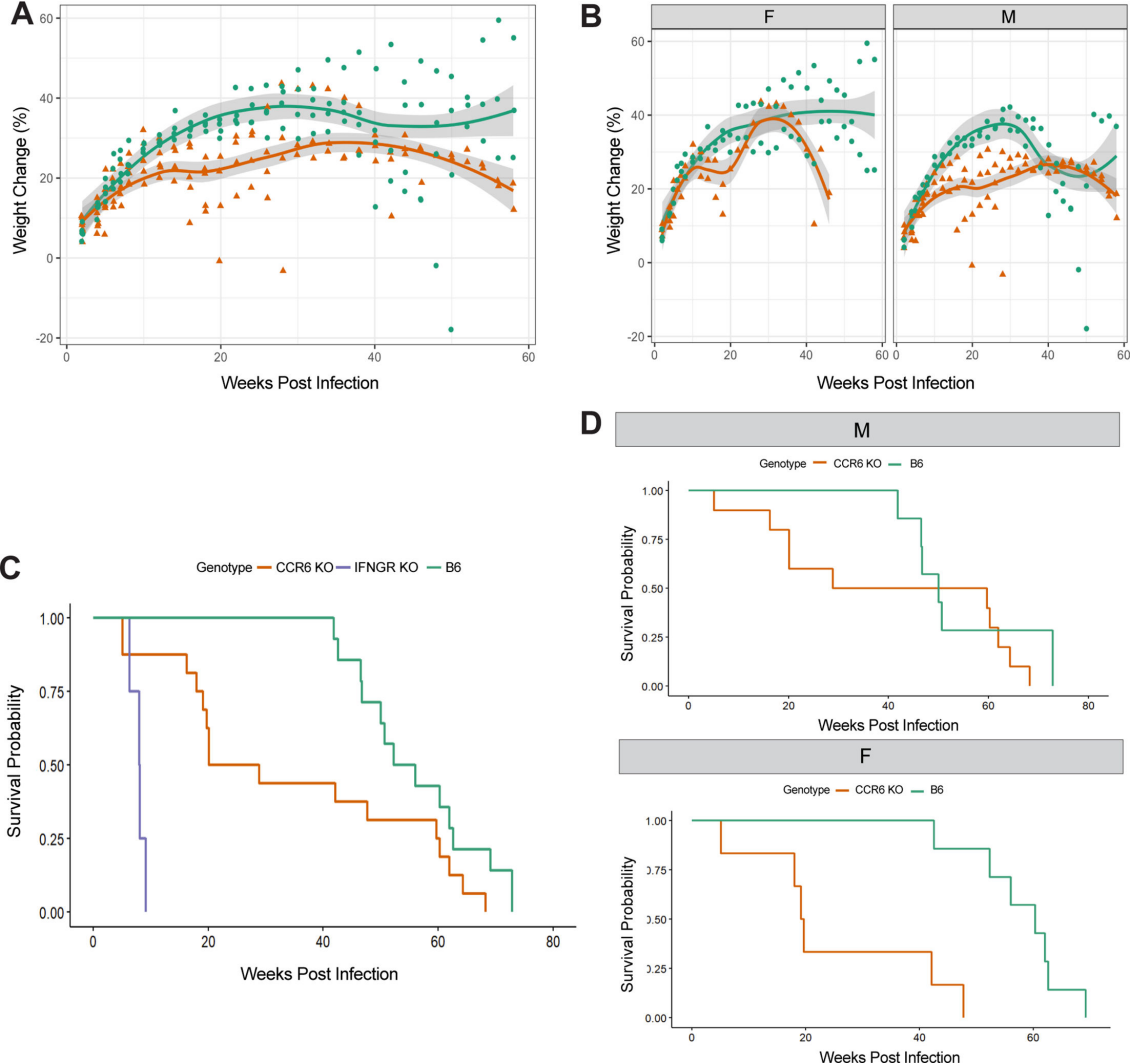
CCR6 KO mice exhibit increased *M.tb* susceptibility and higher post-infection mortality risk. Mice were challenged with *M.tb* H37Rv and weighed bi-weekly for 70 weeks to monitor disease progression. Orange lines represent the average weight of CCR6 KO mice, and green lines represent the average weight of B6 mice (**A**) Kinetics of percent weight change compared to initial body weight at day 0 for the complete cohort. (**B**) Kinetics of percent weight change from initial body weight stratified by sex (*n* = 7−10 mice per genotype and sex). (**C**) Kaplan-Meier survival estimates of *M.tb*-infected CCR6 KO, IFNGR KO, and B6 for the complete cohort. (**D**) Kaplan-Meier survival estimates stratified by sex for *M.tb*-infected CCR6 KO and B6 mice.

Finally, we investigated the impact of CCR6 ablation on long-term *M.tb* infection outcomes. We infected independent cohorts of CCR6 KO and B6 control mice via the aerosol route and evaluated clinical indicators of TB progression over a 70-week period. Interferon-gamma receptor knockout (*Ifngr* KO) mice, which are canonically susceptible to *M.tb* infection (46), were included as controls to confirm successful aerosol infection. Over the study period, animals were weighed bi-weekly to monitor for signs of severe disease and were euthanized at Institutional Animal Care and Use Committee (IACUC)-approved humane endpoints. Compared to B6 controls, infected CCR6 KO mice lost a greater proportion of their initial body weight on average and did not recover within the observation period (Fig. 4A). Upon further analysis, we discovered that this wasting phenotype was present only in female CCR6 KO mice after 30 weeks of infection (Fig. 4B), whereas the body weight of male CCR6 KO mice steadily increased throughout the observation period. Similarly, CCR6 ablation was associated with a significant overall reduction in survival (Fig. 4C; *P* = 0.03), where the increased risk of mortality following infection was also exclusive to female CCR6 KO mice (Fig. 4D, *P* = 6e^−4^ in females, *P* = 0.3 in males). Collectively, these data suggest that CCR6 is an important component of host resistance to *M.tb*, particularly in females.

## DISCUSSION

Genetic variation in human *CCR6* has been implicated in modulating disease severity across several inflammatory diseases. Genome-wide association studies in cohorts with rheumatoid arthritis, primary biliary cholangitis, and several other inflammatory conditions have identified significant associations between *CCR6* polymorphisms and disease severity (47–49). Consistent with these findings, we report that CCR6 ablation *in vivo* enhances TB severity, particularly in female mice.

Research on chronic TB infections in humans (50) and NHPs (27) indicates that the majority of TB-specific CD4^+^ T cells also express CCR6. Nevertheless, the role of CCR6 during virulent mycobacterial infection remains undetermined. Using a mouse model with whole-animal CCR6 ablation, we have determined that CCR6 is necessary for restricting *M.tb*, likely through the promotion of an effective adaptive immune response. Our findings indicate that CCR6 ablation impacts bacterial burden following the onset of adaptive immunity at 4 weeks post-infection, especially in female mice. We also observed necrotic lesions in CCR6 KO mice starting at 4 weeks post-infection, which by 15 weeks progressed to human-like granulomas with fully encapsulated necrotic cores in some animals. These mature granulomas had abundant CD4^+^ T cells in their cellular cuffs that surrounded a necrotic caseum rich in the neutrophil marker Ly-6G. Along with their regular dispersal throughout the cuff, CD4^+^ T cells aggregated with other cells of lymphocyte morphology in some regions, which may represent satellites of tertiary lymphoid tissue. Immune profiling of infected lungs revealed elevated cytokine and chemokine expression in CCR6 KO mice, with high IL-17 levels being a prominent feature of *M.tb* infection in the absence of CCR6. Furthermore, we observed increased mortality risk in female CCR6 KO mice that correlated with an increased bacterial burden.

Our observation of elevated IL-17 levels suggests that dysregulation of T cell signaling likely underlies the disease susceptibility phenotype in CCR6 KO mice. One explanation could be that there is a disruption in CCR6-dependent recruitment of DCs (51, 52) to the lung upon infection and subsequently an ineffective stimulation of T cell responses. However, Chiu and colleagues (53) have demonstrated that CCR6 ablation did not impair DC recruitment to the lung upon stimulation with mycobacterial antigen, indicating that T cell priming likely remains intact. It is also possible that loss of CCR6 in T cells causes a defect in the balance of T cell subsets at sites of infection, resulting in elevated IL-17. In other contexts, CCR6 expression by T cells promotes the recruitment of certain disease-relevant CD4^+^ populations, such as Th17 cells and regulatory T cells (Tregs) (54). Indeed, various studies have shown that CD4^+^ T cells with elevated CCR6 expression migrate to the lungs during *M.tb* infection in humans (28, 50), NHPs (27), and mice (32). Tregs are recruited to the airways during *M.tb* infection in NHPs (55), as well as in mice where they transiently increase susceptibility at early time points by suppressing T cell effector functions (56–58). Gamma-delta (γ/δ) T cells have previously been identified as the primary producers of IL-17 during murine TB (59), and γ/δ T cell IL-17 production can be suppressed by Tregs (60, 61). Importantly, a study of mycobacterial infection in CCR6 KO mice found that CCR6 ablation did not affect γ/δ T cell recruitment to infected lungs (26). This suggests that in CCR6 KO mice, γ/δ T cells that are recruited to the site of infection independently of CCR6 may produce elevated levels of IL-17 in the absence of CCR6-dependent recruitment of suppressive Treg populations.

IL-17 is well-recognized for its role in supporting the adaptive immune response to mycobacterial infection (62–64). It has been previously demonstrated that IL-17-producing CD4^+^ T cells constitute the predominant CCR6-expressing population in both experimentally induced Th17 states and an experimental animal model of autoimmune disease (65, 66). A significant mechanism by which IL-17 can impact the immune response to mycobacteria is by enhancing neutrophil recruitment to the site of infection (67, 68). However, it is crucial to maintain balanced neutrophil levels during chronic TB, and dysregulated IL-17 production can destabilize this balance. In mice, IL-17-mediated neutrophilia is associated with chronic lung inflammation and increased mortality (69, 70). We found an abundance of Ly-6G protein in the acellular caseum of CCR6 KO granulomas, indicating substantial neutrophil recruitment and death within their necrotic cores. Our data support a model in which elevated neutrophil chemoattractants and subsequent enhanced neutrophil recruitment result from IL-17 imbalance in CCR6 KO animals. Future in-depth immunological studies on the residency of T cell and neutrophil populations within CCR6 KO *M.tb* lesions will be needed to evaluate the impact of CCR6 on granuloma cellular constituency.

Furthermore, studies on immune cell trafficking and responses following *M.tb* infection would provide insights into granuloma formation in the context of CCR6 ablation. Necrotic granuloma formation is a hallmark of chronic TB disease in humans and has been studied extensively as a determinant of pathogenesis using various animal models, including NHPs and zebrafish (15, 39, 71, 72). Classical necrotic lesions are largely absent in B6 and other common laboratory mouse strains during *M.tb* infection (73, 74). Mechanistically dissecting the precise role of CCR6 in necrotic granuloma formation will be an important step in understanding the complex immunological signaling that coordinates the granulomatous and adaptive immune responses during *M.tb* infection. Although our observation of necrotic granulomas in *M.tb*-infected CCR6 KO mice is not unprecedented in the context of murine TB research (75–77), it offers a compelling new model for investigating the role of granulomas in TB pathogenesis.

An intriguing aspect of the disease pathology we observed in CCR6 KO mice is the elevated mortality rate in female mice, as male B6 mice typically have higher mortality rates during *M.tb* infection (78, 79). Interestingly, female B6 mice have higher numbers of γ/δ T cells and IL-17 production in their lungs during *M.tb* infection compared to males (80), which may contribute to the sex-dependent differences in disease progression and mortality that we observed. Limited studies have demonstrated a connection between CCR6 and sex-specific responses. These include identified roles for CCR6 in human reproduction, in relation to sperm motility and chemotaxis (81). Interestingly, a correlative relationship has also been reported with experimental autoimmune encephalomyelitis (EAE), a condition induced by the injection of heat-killed *M.tb* into mice, where CCR6 is a prominent EAE-associated gene in male mice (82). Sex is a significant and well-documented mediator of immune responses (83, 84), and this study highlights the importance of gene-specific observations in the context of sex-based biology. Future research should also consider the potential effects of other variables on the host response to *M.tb* infection such as host genetics (85), age (86), and microbiome (87, 88).

## MATERIALS AND METHODS

### Mouse strains and infection with *M.tb*

C57BL/6J (#000664), B6.129P2-*Ccr6^tm1Dgen^*/J (CCR6 KO; #005793), and B6.129S7-*Ifngr1^tm1Agt^*/J (IFNGR KO; #003288) mice were purchased from the Jackson Laboratory. CCR6 KO mice, which were originally purchased from the Jackson Laboratory and maintained on a C57BL/6J (B6) genetic background, were subsequently bred at Duke. All mice were housed in a specific-pathogen-free facility under standard conditions (12 h light/dark, food and water *ad libitum*). Male and female mice were infected between 8 and 12 weeks of age with *M.tb* H37Rv. *M.tb* strains used for *in vivo* infection were verified to be positive for the essential virulence factor, phthiocerol dimycocerosate. *M.tb* was cultured in 7H9 media supplemented with 10% oleic acid-albumin-dextrose-catalase (OADC) enrichment (Middlebrook), 0.2% glycerol, and 0.05% Tyloxapol (Fisher). Prior to infections, *M.tb* cultures were washed and resuspended in phosphate-buffered saline containing 0.05% Tween 80 (PBS-T). Bacterial aggregates were then broken into single cells using a blunt needle before diluting to the desired concentration for infection. An inoculum between 90 and 200 colony-forming units (CFU) was delivered by an aerosol-generating Glas-Col chamber. To determine the inoculation dose, groups of four mice were euthanized at 24 h post-infection, and CFU were enumerated from lung homogenates, as described below.

### Bacterial burden quantification

To quantify CFU, mice were euthanized in accordance with IACUC-approved endpoints by overdose of isoflurane (Covetrus), and the lungs and spleens were aseptically removed. For enumeration of viable bacteria, organs were individually homogenized in PBS-T by bead beating (MP Biomedical), and 10-fold dilutions were plated on 7H10 agar (Middlebrook) plates containing 10% OADC enrichment (Middlebrook) and 50 µg/mL carbenicillin, 10 µg/mL amphotericin B, 25 µg/mL polymyxin B, and 20 µg/mL trimethoprim (Sigma). Plates were incubated at 37°C for 21 days, and individual *M.tb* colonies were enumerated to calculate CFU.

### Quantification of cytokines in tissue homogenate

Following homogenization, infected lung samples were centrifuged through 0.2 µm filters to remove cellular debris. Thirty-two cytokines and chemokines were quantified via Discovery Assay (Eve Technology; MD31). In this assay, fluorescence intensity values were measured for all samples alongside a standard curve to estimate the expected concentrations for each cytokine. Observed cytokine concentration values (reported in pg/mL) were then interpolated for each sample and cytokine using the standard curve and fluorescence intensity values. From this panel, IL-10, IL-12p40, IL-12p70, and IL-13 fell below the detectable limit for all samples and were excluded from further analysis.

### Lung pathology and estimation of inflammation

At 2, 3, and 4 weeks post-infection, one lung lobe from each mouse was collected for histology. Each lobe was fixed in 10% neutral-buffered formalin (VWR) for 24–48 h, then washed 3 times with 70% ethanol. Lungs were submitted to the Duke BioRepository and Precision Pathology Center (BRPC) core, where they were paraffin-embedded, sectioned at 5 µm, and stained with hematoxylin and eosin (H&E). Each whole-lung section was scanned at either 10× or 40× using an Echo Revolution microscope. Whole-slide images (at 40× magnification) were analyzed using Aiforia Create (Version 6.2; Aiforia Technologies Plc, Helsinki, Finland), a cloud-based AI platform designed for training, deploying, and validating pathology image analysis models (89–91). A total of 153 whole-slide images of mouse lung tissue were used for AI model development. Out of the 153 images, 133 were used for model training, and 20 images were left out of the training set for AI model validation purposes. Validation was performed according to a three-point grading system. Four individual validators gave scores for the AI model analysis results according to how closely, in their opinion, the AI model captured the relevant features. The analytical scheme is represented in Fig. S2. For preparation of whole-lung histology samples from long-term experiments, lungs were first perfused with 3 mL of 10% neutral-buffered formalin through the trachea using a 22-gauge needle before being placed in 10% neutral-buffered formalin for 48 h and processed as described above.

### Immunofluorescence of mouse tissue

In total, 5 µm-thick FFPE tissue sections of *M.tb*-infected mouse lung were processed and stained as previously described (92). This study used primary antibodies including rabbit anti-mouse CD4 monoclonal (D7D2Z) antibody (#25229, Cell Signaling) diluted 1:200 and PE-conjugate rat anti-mouse Ly-6G monoclonal (1A8) antibody (#127607, BioLegend) diluted 1:100. Secondary staining for CD4 was conducted using goat anti-rabbit IgG (H+L) Alexa Fluor 647 conjugate (#A21245, Thermo Fisher) diluted 1:500. Slides were mounted with DAPI Fluoromount-G (#0100-20, Southern Biotech) and imaged using a Zeiss LSM 800 spinning-disk confocal microscope.

### Statistical analysis

Hypothesis testing and data visualization were performed using R statistical software (version 4.3.1). Hypothesis testing for differences in bacterial burden, lung damage, and cytokine levels by host genotype was performed by two-way analysis of variance (ANOVA) and Tukey’s *post hoc* test. Differences in survival by host genotype were estimated using Kaplan-Meier analysis in the R package survminer, and statistical significance was assessed via Mantel-Cox hypothesis testing.

## SUPPLEMENTAL MATERIAL

Fig. S1 iai.00154-26-s0001.pdf Granuloma regions with lymphocyte aggregation.

Fig. S2 iai.00154-26-s0002.pdf Cellular infiltration and necrosis in the lung.

ASM does not own the copyrights to Supplemental Material that may be linked to, or accessed through, an article. The authors have granted ASM a non-exclusive, world-wide license to publish the Supplemental Material files. Please contact the corresponding author directly for reuse.
