# Supplementary material for: CCR6 is essential for effective immunity against Mycobacterium tuberculosis infection in mice
Source: Infect Immun. 2026 Apr 30;94(6):e00154-26. doi: 10.1128/iai.00154-26 (PMC13248662; doi:10.1128/iai.00154-26)
Supplement: Fig. S1 — Granuloma regions with lymphocyte aggregation. [file iai.00154-26-s0001.pdf]

Supplemental Figure 1

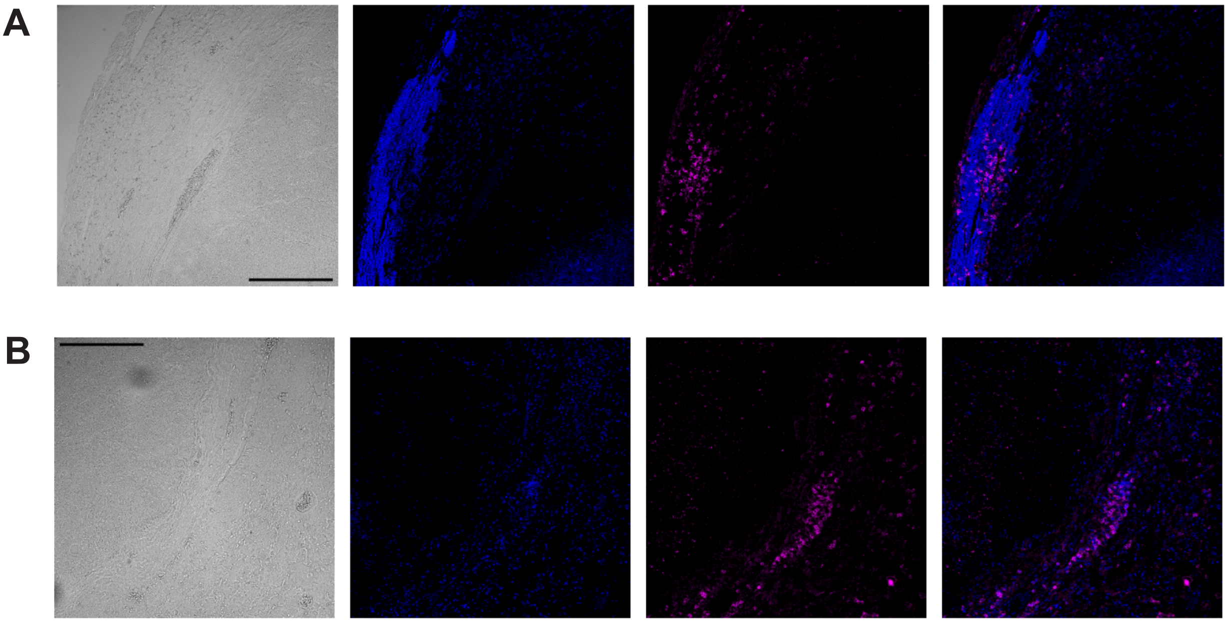

Supplemental Figure 1. Granuloma regions with lymphocyte aggregation. Two regions (A) and (B) of a representative necrotic granuloma from the same sample in Figure 2D showing brightfield and immunofluorescence-stained images of cell nuclei DAPI (blue) and CD4+ T cell (magenta) aggregates in a CCR6 KO mouse M.tb lesion at 20x magnification (scale bar 200 μM)
