# Supplementary material for: CCR6 is essential for effective immunity against Mycobacterium tuberculosis infection in mice
Source: Infect Immun. 2026 Apr 30;94(6):e00154-26. doi: 10.1128/iai.00154-26 (PMC13248662; doi:10.1128/iai.00154-26)
Supplement: Fig. S2 — Cellular infiltration and necrosis in the lung. [file iai.00154-26-s0002.pdf]

Supplemental Figure 2

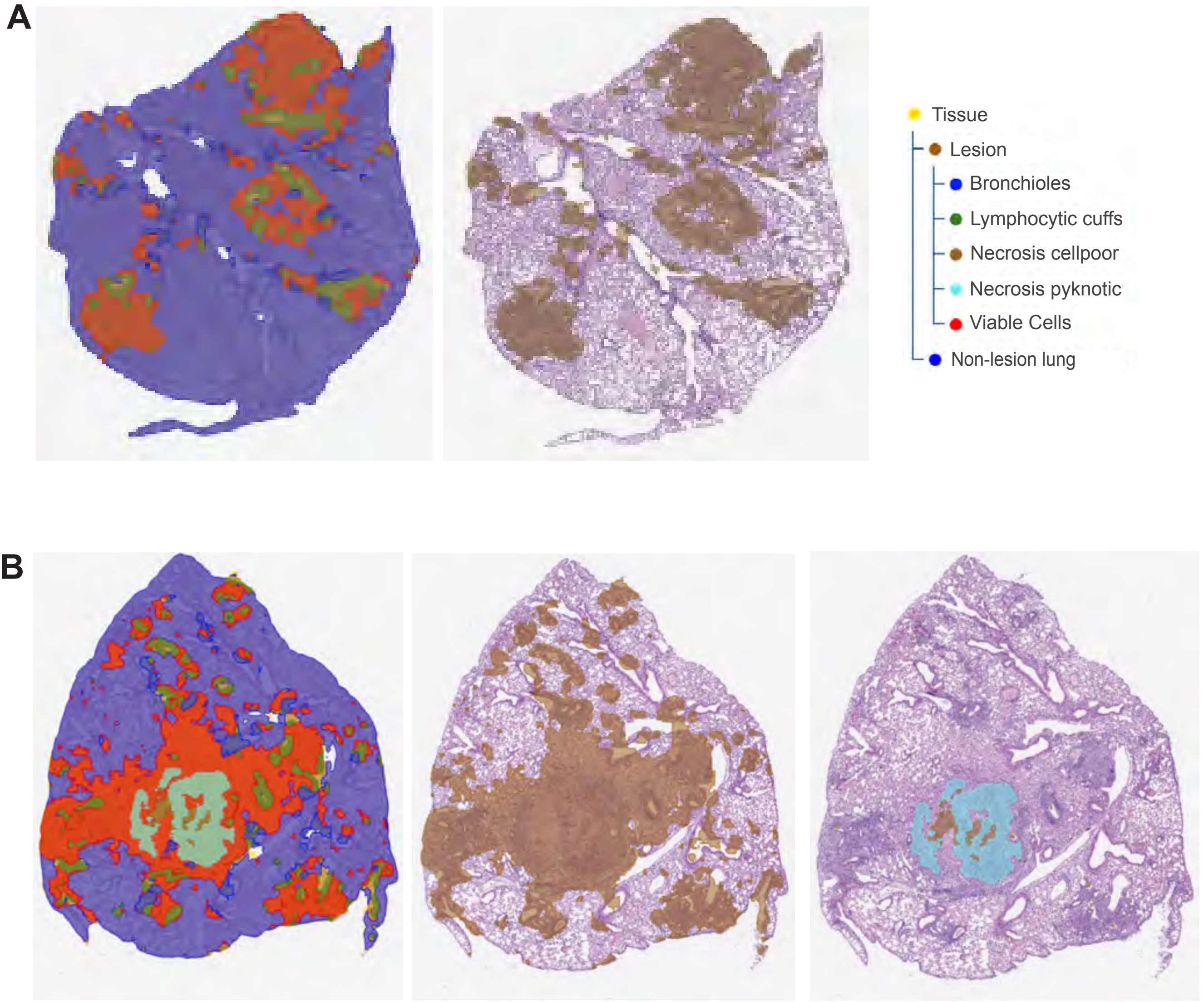

Supplemental Figure 2. Cellular infiltration and necrosis in the lung. An AI-based model analyzed whole-lung scans of H&E-stained sections from both (A) B6 and (B) CCR6 KO male mice. For each image, 3 separate layers were analyzed: tissue vs. background, lesion vs. non-lesion (damage vs. healthy tissue), and lesion sub-regions (bronchioles, lymphocytic cuffs, necrosis cellpoor, necrosis pyknotic, and viable cells). In both A & B the left panel depicts all 3 layers overlapped. The middle panel shows the lesion vs. non-lesion layer. The right panel in A shows the color assignment for each layer and sub-region. The right panel in B only represents the necrosis cellpoor (brown) and necrosis pyknotic (blue) subregions in the lesion layer in CCR6 KO mice, which was not detected in B6 controls.
